# Tetra­chlorido[(diphenyl­phosphino)diphenyl­phosphine oxide-κ*O*]zirconium(IV) benzene monosolvate

**DOI:** 10.1107/S1600536809031882

**Published:** 2009-08-22

**Authors:** Takahiko Ogawa, Yuji Kajita, Hideki Masuda

**Affiliations:** a Department of Applied Chemistry, Nagoya Institute of Technology, Showa-ku, Nagoya 466-8555, Japan

## Abstract

In the title centrosymmetric mononuclear Zr^IV^ compound, [ZrCl_4_{P(O)(C_6_H_5_)_2_P(C_6_H_5_)_2_}_2_]·C_6_H_6_, the central Zr^IV^ ion is coordinated by two O atoms from two symmetry-related (diphenyl­phosphino)diphenyl­phosphine ligands and four Cl atoms in a distorted octahedral geometry with the four Cl atoms in the equatorial positions. The mol­ecule lies about a center of inversion and the benzene solvent mol­ecule about another center of inversion. The P=O bond [1.528 (2) Å] is slightly longer than a typical P=O double bond (average 1.500 ).

## Related literature

For general background to the structure and coordination mode of (diphenyl­phosphino)diphenyl­phosphine (DPDP), see: Ferguson *et al.* (1990 ▶); Kuramshin & Khramov (1983 ▶). For a Zr^IV ^complex with a DPDP ligand, see: Muratova *et al.* (1980 ▶). For comparison P–O bond distances, see: Berners-Price *et al.* (2009 ▶).
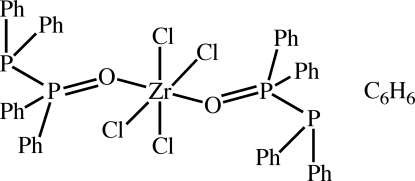


## Experimental

### 

#### Crystal data

[ZrCl_4_(C_24_H_20_OP_2_)_2_]·C_6_H_6_
*M* *_r_* = 1083.81
Triclinic, 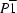

*a* = 9.6073 (3) Å
*b* = 10.1521 (4) Å
*c* = 14.0204 (10) Å
α = 79.027 (13)°
β = 87.269 (13)°
γ = 73.096 (11)°
*V* = 1284.42 (11) Å^3^
*Z* = 1
Mo *K*α radiation
μ = 0.59 mm^−1^
*T* = 173 K
0.30 × 0.15 × 0.13 mm

#### Data collection

Rigaku Mercury diffractometer
Absorption correction: multi-scan (Jacobson, 1998 ▶) *T*_min_ = 0.900, *T*_max_ = 0.927
10324 measured reflections
5669 independent reflections
4497 reflections with *I* > 2σ(*I*)
*R*_int_ = 0.031

#### Refinement

*R*[*F* ^2^ > 2σ(*F* ^2^)] = 0.045
*wR*(*F* ^2^) = 0.151
*S* = 1.02
5669 reflections
296 parameters
H-atom parameters constrained
Δρ_max_ = 1.04 e Å^−3^
Δρ_min_ = −0.81 e Å^−3^

### 

Data collection: *CrystalClear* (Rigaku, 2007 ▶); cell refinement: *CrystalClear*; data reduction: *CrystalStructure* (Rigaku, 2007 ▶); program(s) used to solve structure: *SIR92* (Altomare *et al.*, 1994 ▶); program(s) used to refine structure: *SHELXL97* (Sheldrick, 2008 ▶); molecular graphics: *ORTEP-3* (Farrugia, 1997 ▶); software used to prepare material for publication: *CrystalStructure*.

## Supplementary Material

Crystal structure: contains datablocks global, I. DOI: 10.1107/S1600536809031882/ng2624sup1.cif

Structure factors: contains datablocks I. DOI: 10.1107/S1600536809031882/ng2624Isup2.hkl

Additional supplementary materials:  crystallographic information; 3D view; checkCIF report

## Figures and Tables

**Table 1:** Selected bond lengths (Å)

Zr1—Cl1	2.4450 (9)
Zr1—Cl2	2.4627 (7)
Zr1—O1	2.0820 (18)

**Fig. 1.**
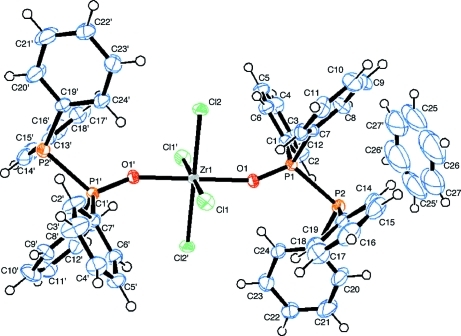
The molecular structure of the title molecule, with the atom-numbering scheme. Displacement ellipsoids are drawn at the 50% probability level. Primed atoms are generated by the symmetry operator ((i) -x + 2, -y, -z + 2; (ii) -x + 1, -y + 1, -z + 1).

**Fig. 2.**
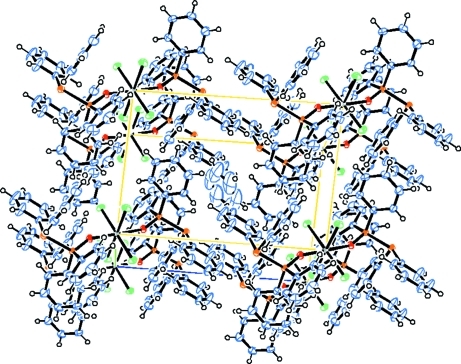
A partial packing diagram of the title compound.

## supplementary crystallographic information

### Comment

The phosphinoyl derivative, (diphenylphosphino)diphenylphosphine (DPDP), has a unique structure and coordination mode (Ferguson 1990 and Muratova *et al.*, 1980). The complex which contains DPDP ligands is very rare and is a novel Zr^IV ^complex with DPDP ligand (Kuramshin & Khramov *et al.*, 1983). The centrosymmetric unit of the title compound, [ZrCl_4_(C_24_H_20_P_2_O)_2_] C_6_H_6_, contains a neutral Zr^IV^ complex and a solvent benzene molecule (Fig. 1). The compound crystallized in the triclinic space group *P*1. In the complex, the Zr^IV^ ion is six-coordinated in a slightly distorted octahedral environment by two O atoms of DPDP ligand and four Cl ions. The P atoms do not coordinate to the Zr^IV^ atom. The Zr—Cl(1) [2.4450 (9) Å] and Zr—Cl(2) [2.4627 (9) Å] bond lengths are slightly different each other (Table 1). The P—O bond length [1.5281 (19) Å] is slightly longer compared with a typical double bond (Berners-Price *et al.*, 2009). The P—P bond length [2.2057 (10) Å] is similar to several P^III^—P^V^ derivatives.

### Experimental

The title compound was prepared by the reaction of ZrCl_4_ (0.03 g, 0.13 mmol) and (diphenylphosphino)diphenylphosphine (0.10 g, 0.26 mmol) in benzene (5 ml). The mixture was filtered and set aside to crystallize at ambient temperature for several days, giving colorless single crystals.

### Refinement

All H atoms were positioned geometrically and refined as riding atoms, with C—H = 0.95 Å and *U*_iso_(H) = 1.2 *U*_eq_(C).

The final difference Fourier map had a peak near the Zr1 atom.

### Crystal data

**Table:**

[ZrCl_4_(C_24_H_20_OP_2_)_2_]·C_6_H_6_	*Z* = 1
*M**_r_* = 1083.81	*F*(000) = 554.00
Triclinic, *P*1	*D*_x_ = 1.401 Mg m^−^^3^
Hall symbol: -P 1	Mo *K*α radiation, λ = 0.71070 Å
*a* = 9.6073 (3) Å	Cell parameters from 3589 reflections
*b* = 10.1521 (4) Å	θ = 3.2–27.5°
*c* = 14.0204 (10) Å	µ = 0.59 mm^−^^1^
α = 79.027 (13)°	*T* = 173 K
β = 87.269 (13)°	Chip, colorless
γ = 73.096 (11)°	0.30 × 0.15 × 0.13 mm
*V* = 1284.42 (11) Å^3^	

### Data collection

**Table:**

Rigaku Mercury diffractometer	4497 reflections with *F*^2^ > 2σ(*F*^2^)
Detector resolution: 7.31 pixels mm^-1^	*R*_int_ = 0.031
ω scans	θ_max_ = 27.5°
Absorption correction: multi-scan (Jacobson, 1998)	*h* = −12→12
*T*_min_ = 0.900, *T*_max_ = 0.927	*k* = −11→13
10324 measured reflections	*l* = −13→18
5669 independent reflections	

### Refinement

**Table:**

Refinement on *F*^2^	0 restraints
*R*[*F*^2^ > 2σ(*F*^2^)] = 0.045	H-atom parameters constrained
*wR*(*F*^2^) = 0.151	*w* = 1/[σ^2^(*F*_o_^2^) + (0.1*P*)^2^] where *P* = (*F*_o_^2^ + 2*F*_c_^2^)/3
*S* = 1.02	(Δ/σ)_max_ < 0.001
5669 reflections	Δρ_max_ = 1.04 e Å^−^^3^
296 parameters	Δρ_min_ = −0.81 e Å^−^^3^

### Special details

**Table:**

Geometry. All e.s.d.'s (except the e.s.d. in the dihedral angle between two l.s. planes) are estimated using the full covariance matrix. The cell e.s.d.'s are taken into account individually in the estimation of e.s.d.'s in distances, angles and torsion angles; correlations between e.s.d.'s in cell parameters are only used when they are defined by crystal symmetry. An approximate (isotropic) treatment of cell e.s.d.'s is used for estimating e.s.d.'s involving l.s. planes.
Refinement. Refinement was performed using all reflections. The weighted *R*-factor (*wR*) and goodness of fit (*S*) are based on *F*^2^. *R*-factor (gt) are based on *F*. The threshold expression of *F*^2^ > 2.0 σ(*F*^2^) is used only for calculating *R*-factor (gt).

### Fractional atomic coordinates and isotropic or equivalent isotropic displacement parameters (Å^2^)

**Table:**

	*x*	*y*	*z*	*U*_iso_*/*U*_eq_	
Zr1	1	0	1	0.01849 (13)	
Cl1	1.14353 (9)	0.14927 (8)	1.03401 (6)	0.0305 (2)	
Cl2	0.79704 (8)	0.12426 (8)	1.09344 (6)	0.02711 (18)	
P1	0.79655 (8)	0.24267 (8)	0.80566 (5)	0.01964 (17)	
P2	0.87015 (9)	0.33171 (9)	0.66302 (6)	0.0249 (2)	
O1	0.9163 (2)	0.1442 (2)	0.87498 (15)	0.0212 (4)	
C1	0.6603 (3)	0.1623 (3)	0.7861 (2)	0.0239 (6)	
C2	0.6455 (4)	0.1237 (4)	0.6987 (2)	0.0404 (9)	
C3	0.5388 (4)	0.0607 (5)	0.6859 (3)	0.0509 (11)	
C4	0.4473 (4)	0.0343 (4)	0.7626 (2)	0.0377 (8)	
C5	0.4620 (3)	0.0716 (3)	0.8512 (2)	0.0346 (7)	
C6	0.5688 (3)	0.1353 (3)	0.8631 (2)	0.0301 (7)	
C7	0.7107 (3)	0.3971 (3)	0.8540 (2)	0.0245 (6)	
C8	0.5776 (3)	0.4851 (3)	0.8158 (2)	0.0339 (7)	
C9	0.5195 (4)	0.6137 (4)	0.8442 (3)	0.0471 (10)	
C10	0.5940 (4)	0.6522 (4)	0.9110 (3)	0.0482 (10)	
C11	0.7256 (4)	0.5653 (3)	0.9496 (2)	0.0401 (9)	
C12	0.7856 (3)	0.4366 (3)	0.9215 (2)	0.0288 (7)	
C13	0.9801 (3)	0.4239 (3)	0.7122 (2)	0.0284 (7)	
C14	0.9168 (4)	0.5667 (3)	0.7125 (2)	0.0375 (8)	
C15	0.9878 (5)	0.6405 (4)	0.7568 (3)	0.0476 (10)	
C16	1.1218 (5)	0.5752 (4)	0.7984 (3)	0.0513 (11)	
C17	1.1876 (4)	0.4354 (4)	0.7969 (3)	0.0484 (10)	
C18	1.1170 (4)	0.3584 (4)	0.7553 (2)	0.0358 (8)	
C19	1.0058 (3)	0.1844 (3)	0.6233 (2)	0.0276 (6)	
C20	1.0628 (5)	0.2151 (4)	0.5313 (3)	0.0552 (12)	
C21	1.1635 (5)	0.1132 (5)	0.4917 (3)	0.0686 (15)	
C22	1.2062 (4)	−0.0226 (4)	0.5424 (2)	0.0423 (9)	
C23	1.1486 (4)	−0.0549 (4)	0.6320 (2)	0.0410 (9)	
C24	1.0484 (4)	0.0476 (3)	0.6725 (2)	0.0358 (8)	
C25	0.4088 (12)	0.5582 (15)	0.5682 (6)	0.165 (7)	
C26	0.5127 (14)	0.6167 (11)	0.5268 (8)	0.167 (5)	
C27	0.6047 (12)	0.5579 (14)	0.4560 (7)	0.171 (6)	
H1	0.7090	0.1403	0.6467	0.049*	
H2	0.5285	0.0358	0.6252	0.061*	
H3	0.3746	−0.0093	0.7543	0.045*	
H4	0.3994	0.0537	0.9034	0.041*	
H5	0.5794	0.1603	0.9237	0.036*	
H6	0.5267	0.4573	0.7704	0.041*	
H7	0.4292	0.6746	0.8179	0.056*	
H8	0.5540	0.7399	0.9307	0.058*	
H9	0.7753	0.5936	0.9954	0.048*	
H10	0.8763	0.3765	0.9477	0.035*	
H11	0.8247	0.6134	0.6823	0.045*	
H12	0.9430	0.7368	0.7582	0.057*	
H13	1.1700	0.6262	0.8285	0.061*	
H14	1.2819	0.3914	0.8245	0.058*	
H15	1.1616	0.2615	0.7562	0.043*	
H16	1.0318	0.3079	0.4953	0.066*	
H17	1.2033	0.1364	0.4296	0.082*	
H18	1.2752	−0.0933	0.5153	0.051*	
H19	1.1776	−0.1486	0.6668	0.049*	
H20	1.0088	0.0235	0.7345	0.043*	
H21	0.3452	0.6006	0.6148	0.199*	
H22	0.5231	0.6982	0.5461	0.200*	
H23	0.6756	0.6003	0.4258	0.205*	

### Atomic displacement parameters (Å^2^)

**Table:**

	*U*^11^	*U*^22^	*U*^33^	*U*^12^	*U*^13^	*U*^23^
Zr1	0.0175 (2)	0.0156 (2)	0.0215 (2)	−0.00302 (15)	0.00179 (15)	−0.00429 (15)
Cl1	0.0292 (4)	0.0249 (3)	0.0416 (4)	−0.0116 (3)	−0.0023 (3)	−0.0096 (3)
Cl2	0.0237 (3)	0.0243 (3)	0.0309 (4)	−0.0023 (3)	0.0078 (3)	−0.0082 (3)
P1	0.0202 (3)	0.0174 (3)	0.0200 (3)	−0.0032 (2)	0.0018 (2)	−0.0041 (2)
P2	0.0277 (4)	0.0234 (4)	0.0211 (3)	−0.0051 (3)	0.0007 (3)	−0.0013 (3)
O1	0.0228 (10)	0.0185 (9)	0.0204 (10)	−0.0038 (8)	−0.0003 (8)	−0.0020 (8)
C1	0.0240 (14)	0.0194 (14)	0.0268 (15)	−0.0042 (12)	0.0027 (12)	−0.0045 (12)
C2	0.043 (2)	0.063 (2)	0.0286 (17)	−0.032 (2)	0.0102 (15)	−0.0183 (17)
C3	0.057 (2)	0.075 (3)	0.042 (2)	−0.042 (2)	0.0090 (19)	−0.029 (2)
C4	0.0338 (18)	0.044 (2)	0.042 (2)	−0.0189 (17)	−0.0023 (15)	−0.0118 (17)
C5	0.0272 (16)	0.0403 (19)	0.0376 (19)	−0.0138 (15)	0.0068 (14)	−0.0054 (15)
C6	0.0313 (17)	0.0321 (17)	0.0274 (16)	−0.0106 (14)	0.0035 (13)	−0.0057 (13)
C7	0.0252 (15)	0.0184 (14)	0.0291 (15)	−0.0046 (12)	0.0053 (12)	−0.0064 (12)
C8	0.0274 (16)	0.0271 (16)	0.045 (2)	−0.0015 (14)	−0.0034 (15)	−0.0106 (15)
C9	0.036 (2)	0.033 (2)	0.063 (2)	0.0057 (16)	0.0071 (19)	−0.0124 (19)
C10	0.060 (2)	0.0254 (18)	0.055 (2)	−0.0007 (18)	0.014 (2)	−0.0178 (17)
C11	0.063 (2)	0.0270 (17)	0.0353 (19)	−0.0181 (18)	0.0053 (17)	−0.0107 (15)
C12	0.0389 (18)	0.0210 (15)	0.0262 (15)	−0.0097 (14)	0.0031 (13)	−0.0026 (12)
C13	0.0317 (16)	0.0284 (16)	0.0237 (15)	−0.0091 (14)	0.0075 (13)	−0.0025 (12)
C14	0.049 (2)	0.0249 (17)	0.0371 (19)	−0.0130 (16)	0.0107 (16)	0.0007 (14)
C15	0.072 (3)	0.0304 (19)	0.047 (2)	−0.026 (2)	0.019 (2)	−0.0107 (17)
C16	0.070 (3)	0.053 (2)	0.049 (2)	−0.041 (2)	0.012 (2)	−0.019 (2)
C17	0.046 (2)	0.060 (2)	0.049 (2)	−0.028 (2)	−0.0003 (19)	−0.015 (2)
C18	0.0355 (18)	0.0371 (19)	0.0354 (18)	−0.0113 (16)	0.0031 (15)	−0.0074 (15)
C19	0.0296 (16)	0.0298 (16)	0.0247 (15)	−0.0089 (13)	0.0026 (12)	−0.0080 (13)
C20	0.077 (3)	0.036 (2)	0.045 (2)	−0.008 (2)	0.030 (2)	−0.0062 (18)
C21	0.085 (3)	0.056 (2)	0.046 (2)	0.003 (2)	0.039 (2)	−0.009 (2)
C22	0.050 (2)	0.041 (2)	0.0311 (18)	−0.0035 (18)	0.0114 (16)	−0.0136 (16)
C23	0.051 (2)	0.0328 (18)	0.0328 (18)	−0.0020 (17)	0.0065 (17)	−0.0076 (15)
C24	0.047 (2)	0.0298 (17)	0.0247 (16)	−0.0032 (15)	0.0104 (15)	−0.0053 (13)
C25	0.142 (9)	0.180 (11)	0.057 (4)	0.105 (9)	0.007 (4)	0.026 (6)
C26	0.168 (10)	0.152 (9)	0.091 (6)	0.074 (8)	−0.027 (6)	0.014 (6)
C27	0.146 (8)	0.169 (10)	0.084 (6)	0.082 (8)	0.016 (5)	0.048 (6)

### Geometric parameters (Å, °)

**Table:**

Zr1—Cl1	2.4450 (9)	C19—C24	1.380 (4)
Zr1—Cl1^i^	2.4450 (9)	C20—C21	1.379 (6)
Zr1—Cl2	2.4627 (7)	C21—C22	1.377 (5)
Zr1—Cl2^i^	2.4627 (7)	C22—C23	1.369 (5)
Zr1—O1	2.0820 (18)	C23—C24	1.386 (5)
Zr1—O1^i^	2.0820 (18)	C25—C26	1.358 (19)
P1—P2	2.2057 (10)	C25—C27^ii^	1.33 (2)
P1—O1	1.5281 (19)	C26—C27	1.399 (16)
P1—C1	1.787 (3)	C2—H1	0.950
P1—C7)	1.788 (3)	C3—H2	0.950
P2—C13	1.833 (4)	C4—H3	0.950
P2—C19	1.833 (3)	C5—H4	0.950
C1—C2	1.382 (5)	C6—H5	0.950
C1—C6	1.397 (4)	C8—H6	0.950
C2—C3	1.391 (7)	C9—H7	0.950
C3—C4	1.392 (6)	C10—H8	0.950
C4—C5	1.390 (6)	C11—H9	0.950
C5—C6	1.394 (6)	C12—H10	0.950
C7—C8	1.393 (4)	C14—H11	0.950
C7—C12	1.397 (5)	C15—H12	0.950
C8—C9	1.387 (5)	C16—H13	0.950
C9—C10	1.382 (7)	C17—H14	0.950
C10—C11	1.379 (5)	C18—H15	0.950
C11—C12	1.389 (5)	C20—H16	0.950
C13—C14	1.400 (4)	C21—H17	0.950
C13—C18	1.397 (4)	C22—H18	0.950
C14—C15	1.388 (7)	C23—H19	0.950
C15—C16	1.366 (6)	C24—H20	0.950
C16—C17	1.379 (6)	C25—H21	0.950
C17—C18	1.390 (7)	C26—H22	0.950
C19—C20	1.392 (5)	C27—H23	0.950
			
Cl(1)···C(11)^iii^	3.525 (4)	H(7)···H(3)^xi^	3.057
Cl(2)···C(4)^iv^	3.562 (4)	H(7)···H(13)^ix^	2.668
Cl(2)···C(5)^iv^	3.585 (4)	H(7)···H(14)^ix^	3.540
C(4)···Cl(2)^iv^	3.562 (4)	H(7)···H(19)^xiii^	3.174
C(5)···Cl(2)^iv^	3.585 (4)	H(7)···H(21)	3.273
C(11)···Cl(1)^iii^	3.525 (4)	H(8)···Cl(1)^iii^	3.494
Cl(1)···H(4)^v^	3.032	H(8)···Cl(2)^vi^	3.258
Cl(1)···H(8)^iii^	3.494	H(8)···C(4)^xi^	3.362
Cl(1)···H(9)^iii^	2.887	H(8)···C(5)^xi^	3.208
Cl(1)···H(12)^iii^	3.318	H(8)···C(6)^vi^	3.398
Cl(2)···H(3)^iv^	2.911	H(8)···H(3)^xi^	3.321
Cl(2)···H(4)^iv^	2.961	H(8)···H(4)^xi^	3.055
Cl(2)···H(7)^vi^	2.927	H(8)···H(4)^vi^	3.535
Cl(2)···H(8)^vi^	3.258	H(8)···H(5)^vi^	2.572
Cl(2)···H(13)^iii^	3.045	H(9)···Cl(1)^iii^	2.887
C(2)···H(17)^vii^	3.413	H(9)···C(16)^iii^	3.110
C(2)···H(18)^vii^	3.110	H(9)···C(17)^iii^	2.899
C(3)···H(17)^vii^	3.277	H(9)···H(10)^iii^	3.588
C(3)···H(18)^vii^	3.282	H(9)···H(13)^iii^	2.949
C(3)···H(23)^ii^	3.560	H(9)···H(14)^iii^	2.582
C(4)···H(8)^viii^	3.362	H(10)···H(9)^iii^	3.588
C(4)···H(15)^ix^	3.020	H(10)···H(13)^iii^	3.143
C(5)···H(8)^viii^	3.208	H(11)···C(20)^x^	3.460
C(5)···H(14)^ix^	3.164	H(11)···C(21)^x^	3.354
C(5)···H(15)^ix^	3.157	H(11)···H(16)^x^	2.879
C(6)···H(4)^iv^	3.442	H(11)···H(17)^x^	2.667
C(6)···H(8)^vi^	3.398	H(11)···H(22)	3.352
C(6)···H(14)^ix^	3.176	H(12)···Cl(1)^iii^	3.318
C(8)···H(14)^ix^	3.237	H(12)···C(23)^xi^	3.490
C(8)···H(21)	3.481	H(12)···C(24)^xi^	3.568
C(9)···H(13)^ix^	3.339	H(12)···H(17)^x^	2.930
C(10)···H(5)^vi^	3.352	H(12)···H(19)^xi^	2.960
C(11)···H(13)^iii^	3.366	H(12)···H(20)^xi^	3.109
C(11)···H(14)^iii^	3.275	H(13)···Cl(2)^iii^	3.045
C(12)···H(13)^iii^	3.465	H(13)···C(9)^v^	3.339
C(13)···H(16)^x^	3.566	H(13)···C(11)^iii^	3.366
C(14)···H(16)^x^	3.027	H(13)···C(12)^iii^	3.465
C(14)···H(17)^x^	3.209	H(13)···H(6)^v^	3.492
C(15)···H(16)^x^	3.479	H(13)···H(7)^v^	2.668
C(15)···H(17)^x^	3.335	H(13)···H(9)^iii^	2.949
C(15)···H(19)^xi^	3.244	H(13)···H(10)^iii^	3.143
C(16)···H(7)^v^	3.426	H(13)···H(19)^xi^	2.912
C(16)···H(9)^iii^	3.110	H(13)···H(21)^v^	3.382
C(16)···H(19)^xi^	3.220	H(14)···C(5)^v^	3.164
C(16)···H(21)^v^	3.293	H(14)···C(6)^v^	3.176
C(17)···H(6)^v^	3.327	H(14)···C(8)^v^	3.237
C(17)···H(9)^iii^	2.899	H(14)···C(11)^iii^	3.275
C(17)···H(21)^v^	3.332	H(14)···H(4)^v^	3.268
C(17)···H(23)^x^	3.371	H(14)···H(5)^v^	3.293
C(18)···H(23)^x^	3.192	H(14)···H(6)^v^	2.665
C(20)···H(11)^x^	3.460	H(14)···H(7)^v^	3.540
C(21)···H(1)^vii^	3.425	H(14)···H(9)^iii^	2.582
C(21)···H(2)^vii^	3.408	H(14)···H(21)^v^	3.424
C(21)···H(11)^x^	3.354	H(14)···H(23)^x^	3.500
C(22)···H(1)^vii^	3.091	H(15)···C(4)^v^	3.020
C(22)···H(2)^v^	3.600	H(15)···C(5)^v^	3.157
C(22)···H(2)^vii^	3.375	H(15)···H(3)^v^	2.914
C(22)···H(3)^v^	3.494	H(15)···H(4)^v^	3.149
C(22)···H(22)^xii^	3.500	H(15)···H(23)^x^	3.233
C(23)···H(3)^v^	3.020	H(16)···C(13)^x^	3.566
C(23)···H(12)^viii^	3.490	H(16)···C(14)^x^	3.027
C(23)···H(21)^xii^	3.506	H(16)···C(15)^x^	3.479
C(24)···H(3)^v^	3.242	H(16)···H(11)^x^	2.879
C(24)···H(12)^viii^	3.568	H(16)···H(23)^x^	3.494
C(25)···H(6)	2.983	H(17)···C(2)^vii^	3.413
C(25)···H(18)^xiii^	3.342	H(17)···C(3)^vii^	3.277
C(26)···H(1)^ii^	3.419	H(17)···C(14)^x^	3.209
C(26)···H(6)	3.481	H(17)···C(15)^x^	3.335
C(26)···H(18)^xiii^	3.138	H(17)···H(1)^vii^	3.071
C(27)···H(6)^ii^	3.518	H(17)···H(2)^vii^	2.827
H(1)···C(21)^vii^	3.425	H(17)···H(11)^x^	2.667
H(1)···C(22)^vii^	3.091	H(17)···H(12)^x^	2.930
H(1)···C(26)^ii^	3.419	H(17)···H(22)^x^	3.566
H(1)···H(17)^vii^	3.071	H(18)···C(2)^vii^	3.110
H(1)···H(18)^vii^	2.397	H(18)···C(3)^vii^	3.282
H(1)···H(22)^ii^	3.424	H(18)···C(25)^xii^	3.342
H(2)···C(21)^vii^	3.408	H(18)···C(26)^xii^	3.138
H(2)···C(22)^ix^	3.600	H(18)···H(1)^vii^	2.397
H(2)···C(22)^vii^	3.375	H(18)···H(2)^vii^	2.762
H(2)···H(17)^vii^	2.827	H(18)···H(21)^xii^	3.046
H(2)···H(18)^vii^	2.762	H(18)···H(22)^xii^	2.675
H(2)···H(22)^ii^	3.190	H(19)···C(15)^viii^	3.244
H(2)···H(23)^ii^	3.595	H(19)···C(16)^viii^	3.220
H(3)···Cl(2)^iv^	2.911	H(19)···H(3)^v^	3.088
H(3)···C(22)^ix^	3.494	H(19)···H(7)^xii^	3.174
H(3)···C(23)^ix^	3.020	H(19)···H(12)^viii^	2.960
H(3)···C(24)^ix^	3.242	H(19)···H(13)^viii^	2.912
H(3)···H(7)^viii^	3.057	H(19)···H(21)^xii^	2.796
H(3)···H(8)^viii^	3.321	H(20)···H(3)^v^	3.452
H(3)···H(15)^ix^	2.914	H(20)···H(12)^viii^	3.109
H(3)···H(19)^ix^	3.088	H(21)···C(8)	3.481
H(3)···H(20)^ix^	3.452	H(21)···C(16)^ix^	3.293
H(4)···Cl(1)^ix^	3.032	H(21)···C(17)^ix^	3.332
H(4)···Cl(2)^iv^	2.961	H(21)···C(23)^xiii^	3.506
H(4)···C(6)^iv^	3.442	H(21)···H(6)	2.756
H(4)···H(4)^iv^	3.226	H(21)···H(7)	3.273
H(4)···H(5)^iv^	2.901	H(21)···H(13)^ix^	3.382
H(4)···H(8)^viii^	3.055	H(21)···H(14)^ix^	3.424
H(4)···H(8)^vi^	3.535	H(21)···H(18)^xiii^	3.046
H(4)···H(14)^ix^	3.268	H(21)···H(19)^xiii^	2.796
H(4)···H(15)^ix^	3.149	H(22)···C(22)^xiii^	3.500
H(5)···C(10)^vi^	3.352	H(22)···H(1)^ii^	3.424
H(5)···H(4)^iv^	2.901	H(22)···H(2)^ii^	3.190
H(5)···H(8)^vi^	2.572	H(22)···H(6)	3.593
H(5)···H(14)^ix^	3.293	H(22)···H(11)	3.352
H(6)···C(17)^ix^	3.327	H(22)···H(17)^x^	3.566
H(6)···C(25)	2.983	H(22)···H(18)^xiii^	2.675
H(6)···C(26)	3.481	H(23)···C(3)^ii^	3.560
H(6)···C(27)^ii^	3.518	H(23)···C(17)^x^	3.371
H(6)···H(13)^ix^	3.492	H(23)···C(18)^x^	3.192
H(6)···H(14)^ix^	2.665	H(23)···H(2)^ii^	3.595
H(6)···H(21)	2.756	H(23)···H(14)^x^	3.500
H(6)···H(22)	3.593	H(23)···H(15)^x^	3.233
H(7)···Cl(2)^vi^	2.927	H(23)···H(16)^x^	3.494
H(7)···C(16)^ix^	3.426		
			
Cl(1)—Zr(1)—Cl(1)^i^	180	C(20)—C(21)—C(22)	119.9 (4)
Cl(1)—Zr(1)—Cl(2)	89.73 (2)	C(21)—C(22)—C(23)	119.6 (3)
Cl(1)—Zr(1)—Cl(2)^i^	90.27 (2)	C(22)—C(23)—C(24)	120.7 (3)
Cl(1)—Zr(1)—O(1)	90.01 (7)	C(19)—C(24)—C(23)	120.5 (3)
Cl(1)—Zr(1)—O(1)^i^	89.99 (7)	C(26)—C(25)—C(27)^ii^	120.6 (10)
Cl(1)^i^—Zr(1)—Cl(2)	90.27 (2)	C(25)—C(26)—C(27)	120.5 (13)
Cl(1)^i^—Zr(1)—Cl(2)^i^	89.73 (2)	C(25)^ii^—C(27)—C(26)	118.9 (12)
Cl(1)^i^—Zr(1)—O(1)	89.99 (7)	C(1)—C(2)—H(1)	119.6
Cl(1)^i^—Zr(1)—O(1)^i^	90.01 (7)	C(3)—C(2)—H(1)	119.6
Cl(2)—Zr(1)—Cl(2)^i^	180	C(2)—C(3)—H(2)	120.2
Cl(2)—Zr(1)—O(1)	89.43 (5)	C(4)—C(3)—H(2)	120.2
Cl(2)—Zr(1)—O(1)^i^	90.57 (5)	C(3)—C(4)—H(3)	119.9
Cl(2)^i^—Zr(1)—O(1)	90.57 (5)	C(5)—C(4)—H(3)	119.9
Cl(2)^i^—Zr(1)—O(1)^i^	89.43 (5)	C(4)—C(5)—H(4)	120.1
O(1)—Zr(1)—O(1)^i^	180	C(6)—C(5)—H(4)	120.1
P(2)—P(1)—O(1)	115.79 (9)	C(1)—C(6)—H(5)	120.0
P(2)—P(1)—C(1)	108.46 (10)	C(5)—C(6)—H(5)	120.0
P(2)—P(1)—C(7)	101.63 (10)	C(7)—C(8)—H(6)	120.1
O(1)—P(1)—C(1)	111.99 (13)	C(9)—C(8)—H(6)	120.1
O(1)—P(1)—C(7)	109.97 (14)	C(8)—C(9)—H(7)	120.3
C(1)—P(1)—C(7)	108.32 (15)	C(10)—C(9)—H(7)	120.3
P(1)—P(2)—C(13)	95.26 (11)	C(9)—C(10)—H(8)	119.4
P(1)—P(2)—C(19)	105.04 (10)	C(11)—C(10)—H(8)	119.5
C(13)—P(2)—C(19)	103.65 (16)	C(10)—C(11)—H(9)	119.9
Zr(1)—O(1)—P(1)	155.63 (13)	C(12)—C(11)—H(9)	119.9
P(1)—C(1)—C(2)	122.1 (2)	C(7)—C(12)—H(10)	120.5
P(1)—C(1)—C(6)	118.3 (2)	C(11)—C(12)—H(10)	120.5
C(2)—C(1)—C(6)	119.6 (3)	C(13)—C(14)—H(11)	119.8
C(1)—C(2)—C(3)	120.7 (3)	C(15)—C(14)—H(11)	119.8
C(2)—C(3)—C(4)	119.7 (4)	C(14)—C(15)—H(12)	119.9
C(3)—C(4)—C(5)	120.1 (4)	C(16)—C(15)—H(12)	119.9
C(4)—C(5)—C(6)	119.8 (3)	C(15)—C(16)—H(13)	119.8
C(1)—C(6)—C(5)	120.1 (3)	C(17)—C(16)—H(13)	119.8
P(1)—C(7)—C(8)	119.6 (2)	C(16)—C(17)—H(14)	119.8
P(1)—C(7)—C(12)	119.5 (2)	C(18)—C(17)—H(14)	119.8
C(8)—C(7)—C(12)	120.4 (3)	C(13)—C(18)—H(15)	120.1
C(7)—C(8)—C(9)	119.8 (3)	C(17)—C(18)—H(15)	120.1
C(8)—C(9)—C(10)	119.4 (3)	C(19)—C(20)—H(16)	119.4
C(9)—C(10)—C(11)	121.1 (3)	C(21)—C(20)—H(16)	119.4
C(10)—C(11)—C(12)	120.2 (4)	C(20)—C(21)—H(17)	120.1
C(7)—C(12)—C(11)	119.0 (3)	C(22)—C(21)—H(17)	120.1
P(2)—C(13)—C(14)	117.1 (2)	C(21)—C(22)—H(18)	120.2
P(2)—C(13)—C(18)	124.0 (2)	C(23)—C(22)—H(18)	120.2
C(14)—C(13)—C(18)	118.8 (3)	C(22)—C(23)—H(19)	119.6
C(13)—C(14)—C(15)	120.4 (3)	C(24)—C(23)—H(19)	119.6
C(14)—C(15)—C(16)	120.2 (3)	C(19)—C(24)—H(20)	119.8
C(15)—C(16)—C(17)	120.3 (5)	C(23)—C(24)—H(20)	119.8
C(16)—C(17)—C(18)	120.5 (4)	C(26)—C(25)—H(21)	119.7
C(13)—C(18)—C(17)	119.8 (3)	C(27)^ii^—C(25)—H(21)	119.7
P(2)—C(19)—C(20)	115.2 (2)	C(25)—C(26)—H(22)	119.7
P(2)—C(19)—C(24)	126.5 (2)	C(27)—C(26)—H(22)	119.8
C(20)—C(19)—C(24)	118.2 (3)	C(25)^ii^—C(27)—H(23)	120.6
C(19)—C(20)—C(21)	121.1 (3)	C(26)—C(27)—H(23)	120.6
			
Cl(1)—Zr(1)—O(1)—P(1)	−126.6 (3)	C(19)—P(2)—C(13)—C(18)	−30.0 (3)
Cl(1)—Zr(1)—O(1)^i^—P(1)^i^	−53.4 (3)	P(1)—C(1)—C(2)—C(3)	179.7 (2)
Cl(1)^i^—Zr(1)—O(1)—P(1)	53.4 (3)	P(1)—C(1)—C(6)—C(5)	−179.4 (2)
Cl(1)^i^—Zr(1)—O(1)^i^—P(1)^i^	126.6 (3)	C(2)—C(1)—C(6)—C(5)	−1.0 (4)
Cl(2)—Zr(1)—O(1)—P(1)	−36.8 (3)	C(6)—C(1)—C(2)—C(3)	1.3 (5)
Cl(2)—Zr(1)—O(1)^i^—P(1)^i^	−143.2 (3)	C(1)—C(2)—C(3)—C(4)	−1.0 (5)
Cl(2)^i^—Zr(1)—O(1)—P(1)	143.2 (3)	C(2)—C(3)—C(4)—C(5)	0.4 (5)
Cl(2)^i^—Zr(1)—O(1)^i^—P(1)^i^	36.8 (3)	C(3)—C(4)—C(5)—C(6)	−0.1 (4)
P(2)—P(1)—O(1)—Zr(1)	−171.5 (3)	C(4)—C(5)—C(6)—C(1)	0.4 (4)
O(1)—P(1)—P(2)—C(13)	−61.58 (14)	P(1)—C(7)—C(8)—C(9)	−171.8 (3)
O(1)—P(1)—P(2)—C(19)	44.08 (17)	P(1)—C(7)—C(12)—C(11)	172.2 (2)
P(2)—P(1)—C(1)—C(2)	19.8 (2)	C(8)—C(7)—C(12)—C(11)	−0.2 (5)
P(2)—P(1)—C(1)—C(6)	−161.7 (2)	C(12)—C(7)—C(8)—C(9)	0.6 (5)
C(1)—P(1)—P(2)—C(13)	171.56 (13)	C(7)—C(8)—C(9)—C(10)	−0.7 (6)
C(1)—P(1)—P(2)—C(19)	−82.77 (16)	C(8)—C(9)—C(10)—C(11)	0.4 (6)
P(2)—P(1)—C(7)—C(8)	72.2 (2)	C(9)—C(10)—C(11)—C(12)	−0.0 (6)
P(2)—P(1)—C(7)—C(12)	−100.3 (2)	C(10)—C(11)—C(12)—C(7)	−0.1 (4)
C(7)—P(1)—P(2)—C(13)	57.55 (15)	P(2)—C(13)—C(14)—C(15)	173.9 (3)
C(7)—P(1)—P(2)—C(19)	163.22 (17)	P(2)—C(13)—C(18)—C(17)	−175.3 (2)
O(1)—P(1)—C(1)—C(2)	−109.2 (2)	C(14)—C(13)—C(18)—C(17)	−0.4 (5)
O(1)—P(1)—C(1)—C(6)	69.2 (2)	C(18)—C(13)—C(14)—C(15)	−1.4 (5)
C(1)—P(1)—O(1)—Zr(1)	−46.5 (4)	C(13)—C(14)—C(15)—C(16)	1.6 (6)
O(1)—P(1)—C(7)—C(8)	−164.6 (2)	C(14)—C(15)—C(16)—C(17)	−0.1 (5)
O(1)—P(1)—C(7)—C(12)	22.9 (3)	C(15)—C(16)—C(17)—C(18)	−1.6 (6)
C(7)—P(1)—O(1)—Zr(1)	74.0 (3)	C(16)—C(17)—C(18)—C(13)	1.9 (6)
C(1)—P(1)—C(7)—C(8)	−41.9 (3)	P(2)—C(19)—C(20)—C(21)	−178.6 (4)
C(1)—P(1)—C(7)—C(12)	145.6 (2)	P(2)—C(19)—C(24)—C(23)	177.4 (3)
C(7)—P(1)—C(1)—C(2)	129.4 (2)	C(20)—C(19)—C(24)—C(23)	1.9 (6)
C(7)—P(1)—C(1)—C(6)	−52.2 (2)	C(24)—C(19)—C(20)—C(21)	−2.5 (7)
P(1)—P(2)—C(13)—C(14)	−98.1 (2)	C(19)—C(20)—C(21)—C(22)	1.8 (8)
P(1)—P(2)—C(13)—C(18)	76.9 (2)	C(20)—C(21)—C(22)—C(23)	−0.3 (7)
P(1)—P(2)—C(19)—C(20)	177.0 (3)	C(21)—C(22)—C(23)—C(24)	−0.4 (7)
P(1)—P(2)—C(19)—C(24)	1.3 (3)	C(22)—C(23)—C(24)—C(19)	−0.5 (6)
C(13)—P(2)—C(19)—C(20)	−83.7 (3)	C(26)—C(25)—C(27)^ii^—C(26)^ii^	−1.8 (15)
C(13)—P(2)—C(19)—C(24)	100.6 (3)	C(27)^ii^—C(25)—C(26)—C(27)	1.8 (16)
C(19)—P(2)—C(13)—C(14)	155.0 (2)	C(25)—C(26)—C(27)—C(25)^ii^	−1.8 (15)

Symmetry codes: (i) −*x*+2, −*y*, −*z*+2; (ii) −*x*+1, −*y*+1, −*z*+1; (iii) −*x*+2, −*y*+1, −*z*+2; (iv) −*x*+1, −*y*, −*z*+2; (v) *x*+1, *y*, *z*; (vi) −*x*+1, −*y*+1, −*z*+2; (vii) −*x*+2, −*y*, −*z*+1; (viii) *x*, *y*−1, *z*; (ix) *x*−1, *y*, *z*; (x) −*x*+2, −*y*+1, −*z*+1; (xi) *x*, *y*+1, *z*; (xii) *x*+1, *y*−1, *z*; (xiii) *x*−1, *y*+1, *z*.
