# Tetra­chlorido[(diphenyl­phosphino)diphenyl­phosphine oxide-κ*O*]zirconium(IV) benzene monosolvate. Corrigendum

**DOI:** 10.1107/S1600536810002230

**Published:** 2010-01-30

**Authors:** Takahiko Ogawa, Yuji Kajita, Hideki Masuda

**Affiliations:** a Department of Applied Chemistry, Nagoya Institute of Technology, Showa-ku, Nagoya 466-8555, Japan

## Abstract

The chemical name of the title compound in the paper by Ogawa, Kajita & Masuda [*Acta Cryst.* (2009), E**65**, m1129] is corrected.

## Abstract 2

Corrigendum to *Acta Cryst.* (2009), E**65**, m1129.

In the paper by Ogawa *et al.* (2009) ▶, the chemical name given in the *Title* should be ‘Tetra­chloridobis[(diphenyl­phos­phino)diphenyl­phosphine oxide-κ*O*]zirconium(IV) benzene monosolvate’.
